# An Efficient Test for Gene-Environment Interaction in Generalized Linear Mixed Models with Family Data

**DOI:** 10.3390/ijerph14101134

**Published:** 2017-09-27

**Authors:** Mauricio A. Mazo Lopera, Brandon J. Coombes, Mariza de Andrade

**Affiliations:** 1 School of Statistics, National University of Colombia, Medellín, Antioquia 050022, Colombia; mauromazo35@gmail.com; 2 Departament of Health Sciences Research, Mayo Clinic, Rochester, MN 55905, USA; coombes.brandon@mayo.edu

**Keywords:** gene-environment interaction, generalized linear mixed model, variance component test, score test, ridge regression, best linear unbiased predictor, family data

## Abstract

Gene-environment (GE) interaction has important implications in the etiology of complex diseases that are caused by a combination of genetic factors and environment variables. Several authors have developed GE analysis in the context of independent subjects or longitudinal data using a gene-set. In this paper, we propose to analyze GE interaction for discrete and continuous phenotypes in family studies by incorporating the relatedness among the relatives for each family into a generalized linear mixed model (GLMM) and by using a gene-based variance component test. In addition, we deal with collinearity problems arising from linkage disequilibrium among single nucleotide polymorphisms (SNPs) by considering their coefficients as random effects under the null model estimation. We show that the best linear unbiased predictor (BLUP) of such random effects in the GLMM is equivalent to the ridge regression estimator. This equivalence provides a simple method to estimate the ridge penalty parameter in comparison to other computationally-demanding estimation approaches based on cross-validation schemes. We evaluated the proposed test using simulation studies and applied it to real data from the Baependi Heart Study consisting of 76 families. Using our approach, we identified an interaction between BMI and the Peroxisome Proliferator Activated Receptor Gamma (*PPARG*) gene associated with diabetes.

## 1. Introduction

Linear mixed models (LMM) have been used to find associations between continuous phenotypes and genetic variants, genes, and gene-environment (GE) interactions in unrelated and related subjects in genome-wide association (GWA) analysis. For unrelated subjects, the analysis can be performed within the generalized linear model framework, however, for related subjects as in the case of family data, one has to include the kinship matrix to take into account the correlation among the relatives for each family. In this paper, we are interested in testing GE interaction for discrete phenotypes. Generalized linear mixed models (GLMM) proposed by Breslow and Clayton [1] is an ideal statistical approach to detect such an interaction with non-continuous phenotypes, because it can treat the familiar effect on the phenotype as a random effect.

Gene-based GE interaction tests have previously been proposed for independent subjects [2,3,4]. While each GE interaction can be tested individually using one single nucleotide polymorphism (SNP) at a time, it is known that single SNP association is not as powerful as the gene-based analysis [2] due to the linkage disequilibrium (LD) present among the SNPs in a gene. Lin et al. [2] proposed a variance component test (VCT) of the interactions by treating the interactions as a random effect. This approach was extended to sequencing data with rare variants [3,5]. To overcome multicollinearity of the coefficients of the genetic markers, Lin et al. [2,3] applied ridge regression penalization of SNP coefficients and estimated the ridge penalty parameter with generalized cross-validation. However, this method is computationally demanding and their final test ignores the tuning of the ridge penalty parameter. Coombes [6] instead proposed treating the genetic coefficients as a random effect in a linear mixed model framework to perform the ridge penalization. This equivalence was initially proposed by Bishop and Tipping [7,8] for Bayesian ridge regression in linear models framework. While this approach was able to incorporate the ridge penalty into the test statistic, it was only developed for a quantitative phenotype [6].

Here, we propose a GLMM GE interaction framework for discrete and continuous phenotypes that treats the coefficients of genetic markers as random effects. Also, because the correlation among relatives cannot be ignored, this modeling framework incorporates the kinship matrix in the GLMM [9]. We test for GE interaction between a set of SNPs and an environment by treating interaction coefficients as random effects using a VCT. Our proposed model includes three random effects: the first for genetic variants, the second for gene-environment interaction, and the third for the inclusion of families. In the methods section, we develop the framework for this model. We present the VCT as proposed by Lin [10] in the presence of several random effects and adapt this VCT to accommodate the interactions [11]. We also prove that the corresponding best linear unbiased predictor (BLUP) in our GLMM model is equivalent to the ridge regression estimator, as proposed by Shen et al. [12]. In our simulations, we show that our model can be efficiently computed using the GMMAT package [13] in R and maintains appropriate type I error as well as sufficient power. Finally, we apply our methodology to test for genetic interactions with BMI associated with diabetes among the Baependi Heart Study [14], which consists of 76 families of varying pedigree size.

## 2. Methods

### 2.1. Generalized Linear Mixed Model

GLMMs have been widely applied in situations where the outcome is discrete and random components are involved in the linear predictor. GLMMs, as proposed by Breslow and Clayton [1], link a response variable $y_{i}$, for i=1,…,n, with vectors $x_{i}$ and $z_{i}$ of explanatory variables associated with the fixed and random effects. Given a *r*-dimensional vector d of random effects, the model is given by $g\left(\mu_{i}^{d}\right)=x_{i}^{T}\beta +z_{i}^{T}d$, where g(.) is known as the link function and $\mu_{i}^{d}=E\left(y_{i}|d\right)$ is the conditional mean. The conditional variance is given by $Var\left(y_{i}|d\right)=\phi \omega_{i}\nu \left(\mu_{i}^{d}\right)$ , with ν(.) is a known function, ϕ is a scale parameter and $\omega_{i}$ are known weights. Denoting the observation vector by $y={\left(y_{1},\;\ldots ,y_{n}\right)}^{T}$ and the design matrices with rows $x_{i}^{T}$ and $z_{i}^{T}$ by X and Z, the general formulation of GLMM is given by

(1) $$g\left(\mu^{d}\right)=X\beta +Zd$$

with $\mu^{d}={\left(\mu_{1}^{d},\;\ldots ,\mu_{n}^{d}\right)}^{T}$, and where d is assumed multivariate normal distributed with mean 0 and covariance matrix D=D(π) depending on an unknown vector π of variance components.

### 2.2. Generalized Linear Mixed Model with Gene-Environment

To set up the GLMM to model GE interaction in families, assume we have a random sample of *N* independent families from a study population, with $n_{i}$ members in the *i*th family such the total number of individuals is $n=\sum_{i=1}^{N}n_{i}$. For the *j*th member in the *i*th family, let $Y_{ij}$ be a discrete or continuous response variable for the phenotype of interest, $X_{ij}={\left(X_{ij}^{1},\;\ldots ,X_{ij}^{p}\right)}^{T}$, with *p* equal to the number of non-environmental covariates, $G_{ij}={\left(G_{ij}^{1},\;\ldots ,G_{ij}^{q}\right)}^{T}$ with *q* equal to number of observed genotypes for SNPs in a gene, $E_{ij}$ the environmental variable of interest, and $S_{ij}={\left(E_{ij}G_{ij}^{1},\;\ldots ,E_{ij}G_{ij}^{q}\right)}^{T}$ the GE interaction. The GE interaction GLMM for families may be written as

(2) $$\begin{array}{ccc} g\left[E(Y_{ij}|\alpha_{ij})\right] & = & X_{ij}^{T}\beta_{1}+E_{ij}\beta_{2}+G_{ij}^{T}\theta +S_{ij}^{T}\gamma +\alpha_{ij}\\ Var(Y_{ij}|\alpha_{ij}) & = & \phi \omega_{ij}^{-1}\nu \left[E(Y_{ij}|\alpha_{ij})\right]\\ \alpha_{i} & = & {\left(\alpha_{i1},\;\ldots ,\alpha_{in_{i}}\right)}^{T}∼N\left(0,2\sigma^{2}\Phi_{i}\right) \end{array}$$

where g(.) is a monotone known function, $Y_{ij}|\alpha_{ij}$ follows a distribution in the exponential family, ν(.) is a known function, ϕ is a scale parameter that may be known or may need to be estimated, $\omega_{ij}$ are known weights (commonly equal to 1), $\Phi_{i}$ is the kinship matrix, and $\sigma^{2}$ is a parameter to be estimated.

Equation (2) can be rewritten using the Cholesky decomposition of the kinship matrix for the *i*th family $2\Phi_{i}=K_{i}K_{i}^{T}$ and assuming that $\alpha_{i}=K_{i}b_{i}$ with $b_{i}∼N\left(0,\sigma^{2}I_{n_{i}}\right)$, where $I_{n_{i}}$ denotes a $\left(n_{i}\times n_{i}\right)$ identity matrix. Then, the family model is given by

(3) $$\begin{array}{ccc} g\left(\mu_{i}^{b}\right) & = & X_{i}\beta_{1}+E_{i}\beta_{2}+G_{i}\theta +S_{i}\gamma +K_{i}b_{i} \end{array}$$

with $\mu_{i}^{b}=E\left(Y_{i}|b_{i}\right)$, $g\left(\mu_{i}^{b}\right)={\left(g\left(\mu_{i1}^{b}\right),\;\ldots ,g\left(\mu_{in_{i}}^{b}\right)\right)}^{T}$, $X_{i}={\left[X_{i1}\ldots X_{in_{i}}\right]}^{T}$, $G_{i}={\left[G_{i1}\ldots G_{in_{i}}\right]}^{T}$, $E_{i}={\left(E_{i1},\ldots ,E_{in_{i}}\right)}^{T}$, and $S_{i}={\left[S_{i1}\ldots S_{in_{i}}\right]}^{T}$.

To simplify the computational burden in the estimation process, we generalize Equation (3), by redefining its vectors and matrices as: $\mu^{b}=E\left(Y|b\right)$, $X={\left[X_{1}\ldots X_{N}\right]}^{T}$, $G={\left[G_{1}\ldots G_{N}\right]}^{T}$, $E={\left(E_{1},\;\ldots ,E_{N}\right)}^{T}$, $S={\left[S_{1}\ldots S_{N}\right]}^{T}$, $K=\mathrm{diag}\{K_{1}\ldots K_{N}\}$ and $b={\left[b_{1}\ldots b_{N}\right]}^{T}$, and

(4) $$\begin{array}{ccc} g\left(\mu^{b}\right) & = & \tilde{X}\beta +G\theta +S\gamma +Kb \end{array}$$

with $\tilde{X}={\left[X\;E\right]}^{T}$ and $\beta ={\left(\beta_{1}^{T},\beta_{2}\right)}^{T}$. Our goal is to test the null hypothesis $H_{0}:\gamma =0$ that the gene of interest has no GE interaction associated with response.

## 3. Proposed GE Interaction Test

As mentioned by Lin et al. [2], to treat γ as a fixed vector and proceed with a *p* degrees of freedom (DF) score test can result in loss of power to test for interaction. Another common strategy is to use a single SNP analysis of GE interaction, which assumes all SNPs are uncorrelated, however, this is usually not the case. In the majority of cases, the SNPs within a gene are highly correlated, thus, here we propose a test that accounts for correlation among SNPs and has uses less DF than the score test.

Using Equation (4), our proposed test assumes γ is a random vector following an arbitrary distribution with mean 0 and variance $\tau I_{q}$. Thus, a test of the null hypothesis $H_{0}:\tau =0$ is equivalent to testing $H_{0}:\gamma =0$. For simplicity, we assume $\gamma ∼N(0,\tau I_{q})$.

In order to account for LD among SNPs in a gene and avoid estimation issues related to multicollinearity, we use a ridge regression approach to impose a penalty on θ in the PQL proposed for GLMM [1]. However, the selection of a penalty parameter can be computationally demanding [2]. Thus, to expedite and incorporate the selection of a penalty parameter used in our proposed test, we specify $d_{1}=G\theta$, as a random effect in Equation (4), with $\theta ∼N(0,\sigma_{\theta }^{2}I_{q})$. By using this approach, we demonstrate later in Section 3.1, under the null model, the best linear unbiased predictor (BLUP) of $d_{1}$ is equivalent to the ridge regression estimator. Denoting $d_{2}=Kb$ and $d_{3}=S\gamma$, and assuming $d_{1}$, $d_{2}$ and $d_{3}$ to be independent, we can write Equation (4) in the GLMM form as

(5) $$\begin{array}{ccc} g\left(\mu^{d}\right) & = & \tilde{X}\beta +d_{1}+d_{2}+d_{3}\\ & = & \tilde{X}\beta +Zd \end{array}$$

with $Z=\left[I_{n}\;I_{n}\;I_{n}\right]$, where $I_{n}$ is the (n×n) identity matrix and $d={\left(d_{1}^{T},d_{2}^{T},d_{3}^{T}\right)}^{T}∼N\left(0,D\right)$, where $D=\mathrm{diag}\left\{\sigma_{\theta }^{2}\left(GG^{T}\right),\;\sigma^{2}\left(KK^{T}\right),\;\tau \left(SS^{T}\right)\right\}$.

Based on the penalized quasi-likelihood (PQL) [1], Lin [10] developed a VCT for independent subjects in the framework of a GLMM. To test the null hypothesis $H_{0}:\tau =0$, the VCT uses the score statistic

(6) $$\begin{array}{ccc} U_{\tau }\left(\hat{\beta },\hat{\pi }\right) & = & \frac{1}{2}\left\{{\left(\tilde{Y}-\tilde{X}\beta \right)}^{T}\Sigma^{-1}SS^{T}\Sigma^{-1}\left(\tilde{Y}-\tilde{X}\beta \right)-\mathrm{tr}\left(\Sigma^{-1}SS^{T}\right)\right\}{|}_{\hat{\beta },\;\hat{\pi }} \end{array}$$

where $\hat{\beta }$ and $\hat{\pi }$ are the maximum likelihood (ML) estimators for β and $\pi ={\left(\sigma_{\theta }^{2},\sigma^{2},\phi \right)}^{T}$, respectively, under the null model as described in Section 3.1. In addition, $\tilde{Y}=\tilde{X}\beta +d_{1}+d_{2}+\varepsilon$ is the corresponding working vector, where $\varepsilon ∼N(0,\phi W^{-1})$ and $W=\mathrm{diag}\left\{\omega_{ij}/\left[\nu \left(\mu_{ij}^{d}\right)g^{\prime }{\left(\mu_{ij}^{d}\right)}^{2}\right]\right\}$ is calculated under the null model, where $g^{\prime }\left(.\right)$ denotes the derivate of function g(.). The covariance matrix for the working vector $\tilde{Y}$ is given by $\Sigma =\sigma_{\theta }^{2}GG^{T}+\sigma^{2}KK^{T}+\phi W^{-1}$.

Since $SS^{T}$ does not have a block diagonal structure, the score $U_{\tau }\left(\hat{\beta },\hat{\pi }\right)$ cannot be written as a sum of *N* independent random variables, corresponding to the families. Therefore, the asymptotic distribution of $U_{\tau }\left(\hat{\beta },\hat{\pi }\right)$ is not a normal distribution, in contrast to the VCT of Lin [10]. Instead, we follow the approach developed by Zhang and Lin [11], and propose, as score statistic, the first term in Equation (6), which corresponds to the quadratic form

$$U_{\tau }=U_{\tau }\left(\hat{\beta },\hat{\pi }\right)=\frac{1}{2}\left\{{\left(\tilde{Y}-\tilde{X}\beta \right)}^{T}\Sigma^{-1}SS^{T}\Sigma^{-1}\left(\tilde{Y}-\tilde{X}\beta \right)\right\}{|}_{\hat{\beta }.\hat{\pi }}$$

To correct for bias, we use the restricted maximum likelihood (REML) estimators [1] in the GLMM framework to obtain $\hat{\beta }$ and $\hat{\pi }$ under the null hypothesis.

Zhang and Lin [11] showed that under $H_{0}:\tau =0$, $U_{\tau }$ follows approximately a mixture of one degree of freedom, independent chi-square distributions. However, for computational ease, we use the Satterthwaite method [15] to approximate the distribution of $U_{\tau }$ by a scaled chi-square distribution $\kappa \chi_{\xi }^{2}$, where the scale parameter κ and the degrees of freedom ξ can be calculated by equating the mean and variance of $U_{\tau }$ to those of $\kappa \chi_{\xi }^{2}$.

When REML estimates are used to calculate $U_{\tau }$, Zhang and Lin [11] showed that the mean and variance of $U_{\tau }$ can be approximated, respectively, by

$$\mathrm{tr}\left(PSS^{T}\right){|}_{\hat{\pi }}\;\mathrm{and}\;I_{\tau }=\frac{1}{2}\left\{\mathrm{tr}\left(PSS^{T}PSS^{T}\right)-J^{T}M^{-1}J\right\}{|}_{\hat{\beta },\hat{\pi }},\;\mathrm{with}$$

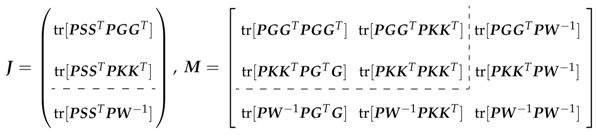

and $P=\Sigma^{-1}-\Sigma^{-1}\tilde{X}{\left({\tilde{X}}^{T}\Sigma^{-1}\tilde{X}\right)}^{-1}{\tilde{X}}^{T}\Sigma^{-1}$.

Dashed lines in J and M represent the cases: (i) ϕ known, implying a (2×1) vector and (2×2) matrix, respectively and (ii) ϕ unknown, implying a (3×1) vector and (3×3) matrix, respectively.

As the mean and variance of $\kappa \chi_{\xi }^{2}$ are given by κξ and $2\kappa^{2}\xi$, respectively, we obtain the equations $\mathrm{tr}(\hat{P}SS^{T})=\kappa \xi$ and $I_{\tau }=2\kappa^{2}\xi$, where $\hat{P}$ denotes the matrix P evaluated in $\hat{\pi }$. By solving these equations, we demonstrate that $\kappa =I_{\tau }/\left[2\mathrm{tr}\left(\hat{P}SS^{T}\right)\right]$ and $\xi =2{\left[\mathrm{tr}(\hat{P}SS^{T})\right]}^{2}/I_{\tau }$.

Therefore, to test $H_{0}:\tau =0$, we propose the statistic

(7) $$\begin{array}{ccc} T_{\tau }=T_{\tau }\left(\hat{\beta },\hat{\pi }\right) & = & \frac{U_{\tau }\left(\hat{\beta },\hat{\pi }\right)}{\kappa } \end{array}$$

which follows approximately a chi-square distribution with ξ degrees of freedom.

### 3.1. Null Model Estimation

Our proposed score test requires that we first fit the null model. Under the null hypothesis $H_{0}:\tau =0$, Equation (5) becomes

(8) $$\begin{array}{ccc} g\left(\mu^{d_{0}}\right) & = & \tilde{X}\beta +Z_{0}d_{0} \end{array}$$

where $d_{0}={\left(d_{1}^{T},d_{2}^{T}\right)}^{T}$, $Z_{0}=\left[I_{n}\;I_{n}\right]$ and $d_{0}∼N\left(0,D_{0}\right)$ with $D_{0}=\mathrm{diag}\left\{\sigma_{\theta }^{2}\left(GG^{T}\right),\;\sigma^{2}\left(KK^{T}\right)\right\}$.

To estimate the parameters in Equation (8), Breslow and Clayton [1] proposed a Fisher scoring solution that may be expressed as the iterative solution to the system

(9) $$\begin{array}{ccc} \left[\begin{array}{cc} {\tilde{X}}^{T}W\tilde{X} & {\tilde{X}}^{T}WZ_{0}\\ ZW\tilde{X} & \phi D_{0}^{-1}+Z_{0}^{T}WZ_{0} \end{array}\right]\left(\begin{array}{c} \beta \\ d_{0} \end{array}\right) & = & \left(\begin{array}{c} {\tilde{X}}^{T}W\tilde{Y}\\ Z_{0}^{T}W\tilde{Y} \end{array}\right) \end{array}$$

This system is equivalent to the so called Henderson equations [16] for computing the best linear unbiased estimator (BLUE) of β and the best linear unbiased predictor (BLUP) of $d_{0}$.

By re-expressing $d_{0}$ to ${\left(d_{1}^{T},d_{2}^{T}\right)}^{T}$ in Equation (9), we obtain the system

(10) $$\left[\begin{array}{ccc} {\tilde{X}}^{T}W\tilde{X} & {\tilde{X}}^{T}W & {\tilde{X}}^{T}W\\ W\tilde{X} & \frac{\phi }{\sigma_{\theta }^{2}}{\left(GG^{T}\right)}^{-1}+W & W\\ W\tilde{X} & W & \frac{\phi }{\sigma^{2}}{\left(KK^{T}\right)}^{-1}+W \end{array}\right]\left(\begin{array}{c} \beta \\ d_{1}\\ d_{2} \end{array}\right)=\left(\begin{array}{c} {\tilde{X}}^{T}W\tilde{Y}\\ W\tilde{Y}\\ W\tilde{Y} \end{array}\right)$$

This new system is equivalent to using a ridge regression penalization for parameter θ in Equation (4) (see Appendix A for details). Assuming that $\pi ={\left(\sigma_{\theta },\sigma ,\phi \right)}^{T}$ is known, it can be shown that the solution of Equation (10) is given by the following equations:

(11) $$\begin{array}{ccc} \hat{\beta } & = & {\left({\tilde{X}}^{T}\Sigma^{-1}\tilde{X}\right)}^{-1}{\tilde{X}}^{T}\Sigma^{-1}\tilde{Y}\\ {\hat{d}}_{0} & = & \left(\begin{array}{c} {\hat{d}}_{1}\\ {\hat{d}}_{2} \end{array}\right)=DZ^{T}\Sigma^{-1}\left(\tilde{Y}-\tilde{X}\hat{\beta }\right)\\ & = & \left(\begin{array}{c} \sigma_{\theta }^{2}\left(GG^{T}\right)\Sigma^{-1}\left(\tilde{Y}-\tilde{X}\hat{\beta }\right)\\ \sigma^{2}\left(KK^{T}\right)\Sigma^{-1}\left(\tilde{Y}-\tilde{X}\hat{\beta }\right) \end{array}\right) \end{array}$$

with $\Sigma =\sigma_{\theta }^{2}GG^{T}+\sigma^{2}KK^{T}+\phi W^{-1}$. Chen et al. [17] fitted their GLMM by defining, $d_{s}=\left(d_{1}+d_{2}\right)$, and assuming $d_{s}∼N\left(0,\left[\sigma_{\theta }^{2}GG^{T}+\sigma^{2}KK^{T}\right]\right)$. However, the BLUP for $d_{s}$ is identical to the sum of BLUPs in Equation (11). Therefore, we use their PQL-based estimation algorithm implemented in the R package GMMAT (see Chen et al. [17] and Chen and Conomos [13] for details) to estimate our null model. The iterative process uses REML to estimate the variance parameters vector π used in the score statistic Equation (7).

## 4. Simulations

**Figure 1:**
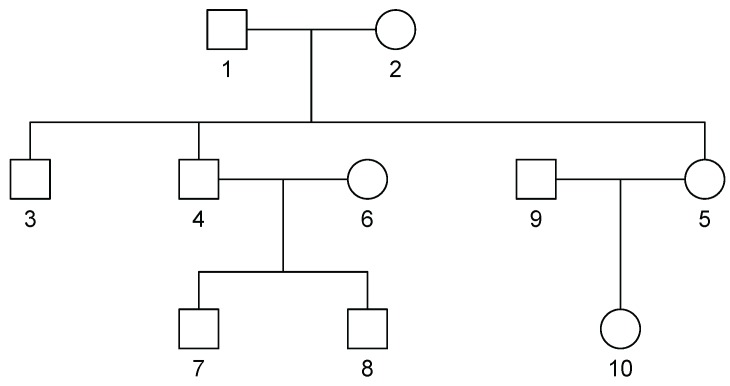
Pedigree for simulated data. Circle = Female, Square = Male.

For our simulations, we used SimPed [18] to generate 100 SNPs (50 independent and 50 in LD) for 1000 independent families with identical pedigree structures of size 10 Figure 1. For each simulation, we randomly sampled without replacement 100 families to obtain a sample of 1000 individuals.

The environment was simulated to be correlated within a family and depend on age and sex of the subject using the following model with parameters chosen to mimic our real data example:

(12) $$\begin{array}{c} E_{ij}=2+0.01Age_{ij}+0.1I\left(Female_{ij}\right)+\gamma_{i}+\varepsilon_{ij} \end{array}$$

where I(·) is the indicator function, $\varepsilon_{i}={\left(\varepsilon_{i1},\;\ldots ,\varepsilon_{i10}\right)}^{T}∼N\left(0,4I_{10}\right)$ where $I_{10}$ is the (10×10) identity matrix, and $\gamma_{i}∼N\left(0,4\right)$.

**Figure 2:**
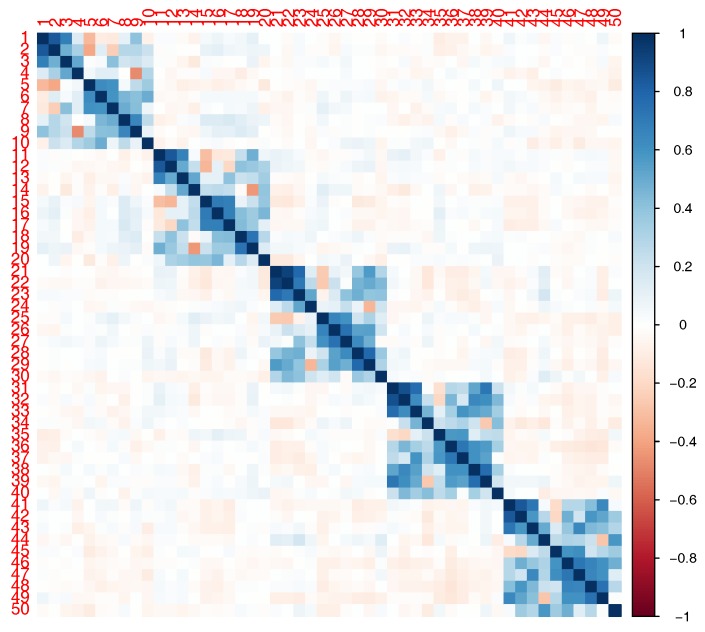
Correlation of the 50 simulated single nucleotide pollymorphisms (SNPs) in linkage disequilibrium (LD). The vertical color line to the right indicates the level of correlation between the SNPs. The dark blue (red) means high positive (high negative) correlation and the light blue (red) means low positive (low negative) correlation.

In our simulations, we considered a SNP-set with 50 independent SNPs or in LD. The correlation structure for the SNPs in LD is shown in Figure 2. Using the R package SimCorMultRes [19], we simulated a correlated binary phenotype dependent on family using the following mean structure:

$$\begin{array}{c} \mathrm{logit}\left[P\left(Y_{ij}=1|Age_{ij},Female_{ij},E_{ij},G1_{ij},G2_{ij}\right)\right]=0.1+0.01Age_{ij}+0.1I\left(Female_{ij}\right)\\ +0.1E_{ij}+0.3G1_{ij}+0.3G2_{ij}+\gamma_{1}\left(G1_{ij}\times E_{ij}\right)+\gamma_{2}\left(G2_{ij}\times E_{ij}\right)+\alpha_{ij} \end{array}$$

with $\alpha_{i}={\left(\alpha_{i1},\;\ldots ,\alpha_{i10}\right)}^{T}∼N\left(0,2\sigma^{2}\Phi_{i}\right)$, where $\Phi_{i}$ is the kinship matrix corresponding to the family pedigree in Figure 1. Given SimCorMultRes only allows for specification of the correlation matrix, $\sigma^{2}$ is set equal to 1. $G1_{ij}$ and $G2_{ij}$ are either independent SNPs (MAF = 0.3 and 0.1) or the first and fifth SNPs with MAF = 0.3 and 0.17 respectively from Figure 2. Note that only the SNPs with a main effect interact with the environment in our model. We generated 10,000 and 1000 datasets to estimate type I error and empirical power, respectively, at an α=0.05 level. Using these datasets, we compared the performances of the score test, MinP test, and our proposed VCT. As previously mentioned, the score test treats γ as a fixed vector and results in a *p* DF test. The null model for this test was estimated as specified in Section 3.1. For the MinP test, which represents a single SNP analysis of GE interaction, we independently model the single SNP-by-environment interaction using Equation (4) where G and S are a vector, rather than a matrix, for a SNP and GE interaction, respectively. For each model, we calculated the *p*-value for the test of interaction and found the minimum *p*-value among all tests. We corrected for multiple testing by multiplying by the number of effective SNPs in the gene [20]. In the case of independence, the number of effective SNPs would be equivalent to the number of SNPs in the gene.

### 4.1. Type I Error

We first compared the empirical type I error of the different methods at 0.05 α-level. To evaluate type I error, we set $\gamma_{1}=\gamma_{2}=0$ and varied the number of SNPs *q* in the gene. The SNPs were either independent or in LD. The empirical type I error rates are shown in Table 1 as well as the mean of the fitted ${\hat{\sigma }}^{2}$, ${\hat{\sigma }}_{\theta }^{2}$, and $\hat{\lambda }=1/{\hat{\sigma }}_{G}^{2}$ parameters from Equation (8) across all simulations. While the variance component for the random effect defined by the kinship matrix stays approximately constant, the penalization term $\hat{\lambda }$ increases as the number of SNPs in the model increases. Thus, like in ridge regression, increasing the number of parameters results in an increased penalization of the model. All of the methods were conservative in our simulations, but as *q* increased, the score test became useless due to the large DF of the test.

### 4.2. Empirical Power for Independent SNPs

**Figure 3:**
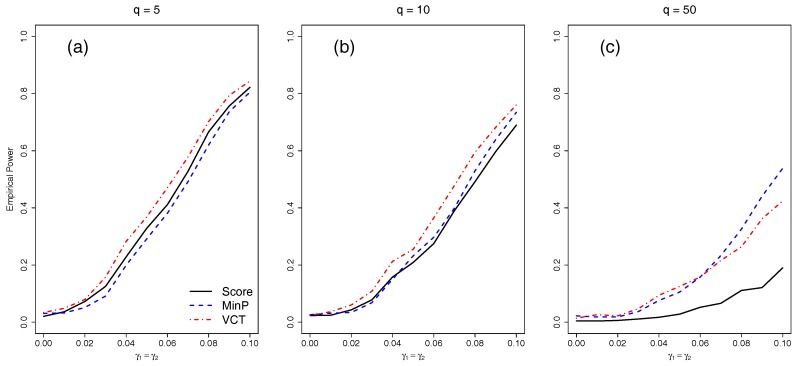
Empirical power at 0.05 α-level of the methods for *q* independent SNPs of which two of the SNPs have a main effect. The same two SNPs have an equal interaction with the environment. (**a**) q=5 independent SNPs; (**b**) q=10 independent SNPs; (**c**) q=50 independent SNPs.

We next compared the empirical power of the different methods with either five, 10, or 50 independent SNPs. We varied the amount of interaction for the two selected SNPs by varying $\gamma_{1}=\gamma_{2}$ from 0 to 0.1 by 0.01. Figure 3 shows that as the number of SNPs in the model increases, each of the methods lose power due to the increase in DF of each test. While the VCT performs best with five or 10 SNPs, the MinP test performs best for 50 SNPs because only two out of 50 SNPs have interaction. The MinP test will always perform best if very few of the SNPs in the set have interaction.

### 4.3. Empirical Power for SNPs in LD

**Figure 4:**
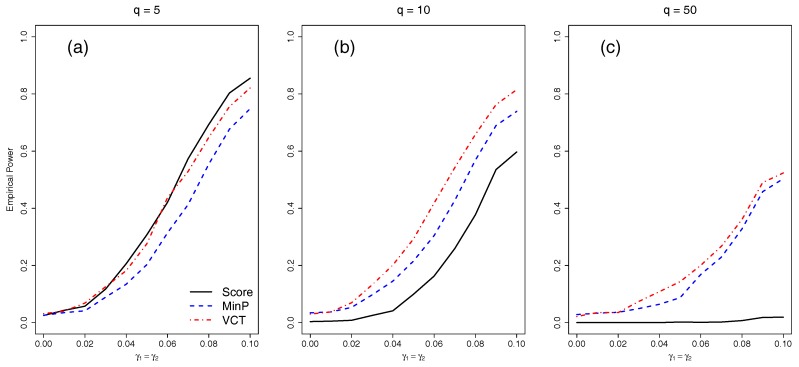
Empirical power at 0.05 α-level of the methods for *q* correlated SNPs of which two of the SNPs have a main effect. The same two SNPs have an equal interaction with the environment. (**a**) q=5 correlated SNPs; (**b**) q=10 correlated SNPs; (**c**) q=50 correlated SNPs.

Finally, we compared the empirical power of the different methods with either five, 10, or 50 SNPs in LD. We varied the amount of interaction for the two selected SNPs by varying $\gamma_{1}=\gamma_{2}$ from 0 to 0.1 by 0.01. Figure 4 shows that as before, each of the methods lose power as *q* increases. The VCT outperforms the MinP test in all scenarios because the SNPs are correlated which the MinP test fails to account for.

## 5. Application to Baependi Data

We use our proposed method to test for GE interactions in the Baependi Heart Study [14] between BMI and three different candidate genes that may be associated with type II diabetes (T2D). The first candidate gene we studied was the Peroxisome-Proliferator-Activated Receptors gamma (*PPARG*) gene, which is a key regulator of adipocyte differentiation and energy balance. Two of the mutations in the *PPARG* gene have been shown to be associated with obesity or diabetes-related phenotypes in different populations [21]. *PPARG2*, the predominantly isoform of *PPARG*, is expressed selectively and at a higher level in adipose tissue, where it modulates the expression of target genes implicated in adipocyte differentiation and glucose homeostasis [22]. Thus, the *PPARG2* gene is a major candidate gene for T2D or obesity, both being complex phenotypes determined by the combination of multiple genetic and environmental factors [23,24]. The second candidate gene studied was the Fat Mass and Obesity associated protein (*FTO*), which confers risk for obesity and BMI. Since obesity is known to be a predisposing factor for the development of T2D, it is not surprising that variants in FTO have been also found in T2D GWAS [25]. The final candidate gene studied was the cyclin-dependent kinase 5 regulatory subunit associated protein 1-like 1 (*CDKAL1*) gene which confers risk for obesity and T2D [26]. In our study, the *PPARG*, *FTO*, and *CDKAL1* genes had 16, 149, and 186 genetic variants genotyped, respectively. However these numbers of SNPs do not represent the number of effective SNPs discussed in Gao et al. [20], that is equivalent of the number of principal components to reach 99.5% of their total variation. Then, the effective number of SNPs associated with the *PPARG*, *FTO*, and *CDKAL1* genes are 10, 93, and 92, respectively. We used Equation (5) to specify a logistic GLMM to test for GE interaction of the aforementioned genes with BMI associated with T2D status (case/control). Our model included age, sex, and the first two principal components of the entire genotype data of Baependi data as covariates. Due to some individuals missing genotype information for some SNPs, the tests of each gene had different sample sizes. Table 2 describes the sample sizes (number of subjects and number of families) for cases and controls included in analysis of each gene.

We use our proposed method to test for GE interactions in the Baependi Heart Study [14] between BMI and three different candidate genes that may be associated with type II diabetes (T2D). The first candidate gene we studied was the Peroxisome-Proliferator-Activated Receptors gamma (*PPARG*) gene, which is a key regulator of adipocyte differentiation and energy balance. Two of the mutations in the *PPARG* gene have been shown to be associated with obesity or diabetes-related phenotypes in different populations [21]. *PPARG2*, the predominantly isoform of *PPARG*, is expressed selectively and at a higher level in adipose tissue, where it modulates the expression of target genes implicated in adipocyte differentiation and glucose homeostasis [22]. Thus, the *PPARG2* gene is a major candidate gene for T2D or obesity, both being complex phenotypes determined by the combination of multiple genetic and environmental factors [23,24]. The second candidate gene studied was the Fat Mass and Obesity associated protein (*FTO*), which confers risk for obesity and BMI. Since obesity is known to be a predisposing factor for the development of T2D, it is not surprising that variants in FTO have been also found in T2D GWAS [25]. The final candidate gene studied was the cyclin-dependent kinase 5 regulatory subunit associated protein 1-like 1 (*CDKAL1*) gene which confers risk for obesity and T2D [26]. In our study, the *PPARG*, *FTO*, and *CDKAL1* genes had 16, 149, and 186 genetic variants genotyped, respectively. However these numbers of SNPs do not represent the number of effective SNPs discussed in Gao et al. [20], that is equivalent of the number of principal components to reach 99.5% of their total variation. Then, the effective number of SNPs associated with the *PPARG*, *FTO*, and *CDKAL1* genes are 10, 93, and 92, respectively. We used Equation (5) to specify a logistic GLMM to test for GE interaction of the aforementioned genes with BMI associated with T2D status (case/control). Our model included age, sex, and the first two principal components of the entire genotype data of Baependi data as covariates. Due to some individuals missing genotype information for some SNPs, the tests of each gene had different sample sizes. Table 2 describes the sample sizes (number of subjects and number of families) for cases and controls included in analysis of each gene.

In Table 3, we report the *p*-values for each method. By comparing the *p*-values with respect to the corresponding α level, only the VCT identifies a significant GE interaction of BMI with *PPARG*. All other tests were non-significant for this gene as well as for other candidate genes.

The variance estimates for families ${\hat{\sigma }}^{2}$ and the ridge penalty ${\hat{\sigma }}_{\theta }^{2}$ are reported in Table 3. In Section 4, we showed that the ridge penalty increases as the number of SNPs increases, however, our results for *PPARG*, *FTO* and *CDKAL1* suggest that λ also depends on the number of subjects. Finally, Table 3 also shows the execution times using the R version 3.3.1 and a processor Intel(R) Core(TM) i5-6500 CPU @ 3.20 GHz with a RAM memory 8.00 GB and operating system 64-bits. Computation times for the VCT and the score test were considerably lower than those for the MinP test. The time to compute each test increased with the increase in number of SNPs in a gene and number of subjects in the analysis.

## 6. Conclusions

We have proposed a variance component score test for testing for interactions between a set of SNPs in a gene and an environmental variable with family data. We specified the interaction coefficients as random variables with common variance and evaluated the null hypothesis that the variance is equal to zero. Given the LD among some SNPs in a gene, we fit the null model assuming the SNP coefficients as random effects and showed that the corresponding BLUP was equivalent to ridge regression estimator. This approach gives a direct estimation for the ridge penalization parameter in comparison with other computationally demanding procedures based on cross validation [2]. We compared, via simulations and a real data application, our approach with the so called MinP test and also with the traditional *q* degrees of freedom score test. The results showed that the proposed test is robust and performs well, with considerable power. Simulations and application presented in this paper were done assuming a binary phenotype and a continuous environmental variable, however, the GLMM admits phenotypes with distribution belonging to the exponential family. These other distributions are currently unexplored in this paper. In addition, it is possible to have multiple environmental factors as well as environmental factors that are discrete. The proposed model can easily incorporate these cases. It is important to note that the proposed model does not account for a possible correlation of the environment among family members. A possible extension of this work is to include in the GLMM a shared household factor by adding a random effect that follows a normal distribution with mean vector zero and with variance the matrix that characterizes household sharing. Finally, using GLMMs can be computationally intensive and may experience convergence issues. In the future, we plan to explore using generalized estimating equations as an alternative approach to testing for interactions in families.

## Figures and Tables

**Table 1:** Type I error at 0.05 α-level for each method depending on the number of SNPs *q* and whether the SNPs are independent or in LD. Two of the SNPs in each scenario have a main effect.

SNPs Category	*q*	${\hat{\sigma }}^{2}$	${\hat{\sigma }}_{\theta }^{2}$	$\hat{\lambda }=1/{\hat{\sigma }}_{G}^{2}$	Score	MinP	VCT
Independent	5	1.247	0.034	29.4	0.020	0.031	0.034
	10	1.240	0.017	58.8	0.023	0.026	0.024
	50	1.222	0.003	333	0.004	0.022	0.014
LD	5	1.243	0.021	47.6	0.025	0.026	0.031
	10	1.239	0.009	111	0.004	0.034	0.030
	50	1.222	0.002	500	0.000	0.028	0.022

**Table 2:** Summary of cases per subjects and families.

Gene	Subjects			Families		
	Control	Cases	Total	Control	Cases	Total
PPARG	845	83	928	43	42	85
FTO	712	71	783	47	38	85
CDKAL1	661	69	730	47	38	85

**Table 3:** Sample size, GLMM parameters, *p*-values and execution times for the analysis of the Baependi dataset.

Gene	SNPs	Total Subjects	GLMM Parameters			Test	*p*-Value	α Level	Time (s)
			${\hat{\sigma }}^{2}$	${\hat{\sigma }}_{\theta }^{2}$	$\hat{\lambda }=1/{\hat{\sigma }}_{G}^{2}$				
						VCT	0.028	0.05	18.420
PPARG	16	928	0.4463	0.0029	344.8276	MinP	0.019 *	0.005	100.261
						Score	0.595	0.05	9.025
						VCT	0.451	0.05	12.958
FTO	149	783	0.3710	0.0033	303.0303	MinP	0.031 *	0.0005	2675.907
						Score	0.992	0.05	6.197
						VCT	0.635	0.05	9.907
CDKAL1	186	730	0.0918	0.0111	90.0901	MinP	0.040 *	0.0005	1755.881
						Score	0.915	0.05	4.257

* Compare MinP test *p*-value with the corresponding corrected α, obtained by dividing 0.05 for the number of effective SNPs (which is equivalent to the number of principal components that reach 99.5% of the their total variation): 10 for *PPARG*, 93 for *FTO* and 92 for *CDKAL1*.

## Abbreviations

The following abbreviations are used in this manuscript:

LMM	Linear Mixed Model
GLMM	Generalized Linear Mixed Model
GE	Gene-Environment
SNP	Single Nucleotide Pollymorphism
BLUP	Best Linear Unbiased Predictor
PPARG	Peroxisome Proliferator Activated Receptor Gamma
GWA	Genome Wide Association
LD	Linkage Disequilibrium
VCT	Variance Components Test
ML	Maximum Likelihood
REML	Restricted Maximum Likelihood
PQL	Penalized Quasi-Likelihood

## Appendix A. Ridge Regression and BLUP Equivalence in GLMM

Using the same notation of Section 2.2, where $d_{2}=Kb$, the Equation (4) under the null hypothesis $H_{0}:\gamma =0$ becomes to

$$g\left(\mu^{d_{2}}\right)=\tilde{X}\beta +G\theta +d_{2}$$

with $\mu^{d_{2}}=E\left(Y|d_{2}\right)$. Following Breslow and Clayton [1], the penalized quasi-likelihood (PQL) for this model is given by

$$\begin{array}{ccc} ql(\beta ,\theta ,\phi_{R},\sigma_{R}) & = & -\frac{1}{2}\log|\frac{\sigma_{R}^{2}}{\phi_{R}}\left(KK^{T}\right)W_{R}+I_{n}|\\ & & +\sum_{i=1}^{N}\sum_{j=1}^{n_{i}}ql_{ij}\left(\beta ,\theta ,\phi_{R};{\tilde{d}}_{2}\right)-\frac{1}{2}{\tilde{d}}_{2}^{T}{\left(\sigma_{R}^{2}KK^{T}\right)}^{-1}{\tilde{d}}_{2} \end{array}$$

with ${\tilde{d}}_{2}$ is choosen to maximize the sum of the last two terms, $W_{R}=\mathrm{diag}\left\{\omega_{ij}/\left[\nu \left(\mu_{ij}^{d_{2}}\right)g^{\prime }{\left(\mu_{ij}^{d_{2}}\right)}^{2}\right]\right\}$ and

$$ql_{ij}\left(\beta ,\theta ,\phi_{R};d_{2}\right)=\int_{Y_{ij}}^{\mu_{ij}^{d_{2}}}\frac{\omega_{ij}\left(Y_{ij}-\mu \right)}{\phi_{R}\nu \left(\mu \right)}d\mu$$

Subindex *R* denotes that the parameters are being estimated under Ridge regression method. We maximize the PQL with respect to β, θ and $d_{2}={\tilde{d}}_{2}$ jointly, obtaining the partial derivates

(A1) $$\begin{array}{ccc} \frac{\partial ql(\beta ,\theta ,\phi_{R},\sigma_{R})}{\partial \beta } & = & \frac{1}{\phi_{R}}{\tilde{X}}^{T}W_{R}\Delta_{R}\left(Y-\mu^{d_{2}}\right)\\ \frac{\partial ql(\beta ,\theta ,\phi_{R},\sigma_{R})}{\partial \theta } & = & \frac{1}{\phi_{R}}G^{T}W_{R}\Delta_{R}\left(Y-\mu^{d_{2}}\right)\\ \frac{\partial ql(\beta ,\theta ,\phi_{R},\sigma_{R})}{\partial d_{2}} & = & \frac{1}{\phi_{R}}W_{R}\Delta_{R}\left(Y-\mu^{d_{2}}\right)-{\left(\sigma_{R}^{2}KK^{T}\right)}^{-1}d_{2} \end{array}$$

with $\Delta_{R}=\mathrm{diag}\left\{g^{\prime }\left(\mu^{d_{2}}\right)\right\}$ (see Chen et al. [17] for details). Ridge regression estimator of θ is obtained by minimizing the function

$$\left[ql\left(\beta ,\theta ,\phi_{R},\sigma_{R}\right)\times \phi_{R}\right]-\frac{1}{2}\lambda \theta^{T}\theta$$

where λ is a penalizing factor and function $ql(\beta ,\theta ,\phi_{R},\sigma_{R})$ is multiplied by $\phi_{R}$ to be consistent with the definition of ridge regression estimator under normality [27]. Therefore, the second line of Equation (A1) becomes

$$\begin{array}{ccc} \frac{\partial ql(\beta ,\theta ,\phi_{R},\sigma_{R})}{\partial \theta } & = & G^{T}W_{R}\Delta \left(Y-\mu^{d_{2}}\right)-\lambda \theta \end{array}$$

and the system of equations is therefore

(A2) $$\begin{array}{ccc} {\tilde{X}}^{T}W_{R}\Delta_{R}\left(Y-\mu^{d_{2}}\right) & = & 0\\ G^{T}W_{R}\Delta_{R}\left(Y-\mu^{d_{2}}\right)-\lambda \theta & = & 0\\ W_{R}\Delta_{R}\left(Y-\mu^{d_{2}}\right)-\frac{\phi_{R}}{\sigma_{R}^{2}}{\left(KK^{T}\right)}^{-1}d_{2} & = & 0 \end{array}$$

and left-multiplying by ${\left(GG^{T}\right)}^{-1}G$ the second line of Equation (A2), we have

(A3) $$\begin{array}{ccc} {\tilde{X}}^{T}W_{R}\Delta_{R}\left(Y-\mu^{d_{2}}\right) & = & 0\\ W_{R}\Delta_{R}\left(Y-\mu^{d_{2}}\right)-\lambda {\left(GG^{T}\right)}^{-1}G\theta & = & 0\\ W_{R}\Delta_{R}\left(Y-\mu^{d_{2}}\right)-\frac{\phi_{R}}{\sigma_{R}^{2}}{\left(KK^{T}\right)}^{-1}d_{2} & = & 0 \end{array}$$

But $\Delta_{R}\left(Y-\mu^{d_{2}}\right)=\tilde{Y}-\tilde{X}\beta -G\theta -d_{2}$, so the Equation (A3) becomes

(A4) $$\begin{array}{ccc} {\tilde{X}}^{T}W_{R}\tilde{X}\beta +{\tilde{X}}^{T}W_{R}G\theta +{\tilde{X}}^{T}W_{R}d_{2} & = & {\tilde{X}}^{T}W_{R}\tilde{Y}\\ \;W_{R}\tilde{X}\beta +\left[W_{R}+\lambda {\left(GG^{T}\right)}^{-1}\right]G\theta +W_{R}d_{2} & = & W_{R}\tilde{Y}\\ W_{R}\tilde{X}\beta +W_{R}G\theta +\left[W_{R}+\frac{\phi_{R}}{\sigma_{R}^{2}}{\left(KK^{T}\right)}^{-1}\right]d_{2} & = & W_{R}\tilde{Y} \end{array}$$

and denoting $d_{1}=G\theta$, we have the matricial representation of Equation (A4)

$$\left[\begin{array}{ccc} {\tilde{X}}^{T}W_{R}\tilde{X} & {\tilde{X}}^{T}W_{R} & {\tilde{X}}^{T}W_{R}\\ W_{R}\tilde{X} & W_{R}+\lambda {\left(GG^{T}\right)}^{-1} & W_{R}\\ W_{R}\tilde{X} & W_{R} & W_{R}+\frac{\phi_{R}}{\sigma_{R}^{2}}{\left(KK^{T}\right)}^{-1} \end{array}\right]\left(\begin{array}{c} \beta \\ d_{1}\\ d_{2} \end{array}\right)=\left(\begin{array}{c} {\tilde{X}}^{T}W_{R}\tilde{Y}\\ W_{R}\tilde{Y}\\ W_{R}\tilde{Y} \end{array}\right)$$

which is equivalent to the Equation (10), where $d_{1}$ is assumed as a normal random effect with mean 0 and variance $\sigma_{\theta }^{2}GG^{T}$ and, also, the penalization factor λ is identical to $\phi /\sigma_{\theta }^{2}$.
